# A Preparation Method of Nano-Pesticide Improves the Selective Toxicity toward Natural Enemies

**DOI:** 10.3390/nano12142419

**Published:** 2022-07-14

**Authors:** Shuo Yan, Na Gu, Min Peng, Qinhong Jiang, Enliang Liu, Zhiqiang Li, Meizhen Yin, Jie Shen, Xiangge Du, Min Dong

**Affiliations:** 1 Department of Plant Biosecurity and MARA Key Laboratory of Surveillance and Management for Plant Quarantine Pests, College of Plant Protection, China Agricultural University, Beijing 100193, China; yanshuo2011@foxmail.com (S.Y.); s20193192665@cau.edu.cn (N.G.); j.yucheng@outlook.com (Q.J.); shenjie@cau.edu.cn (J.S.); duxge@cau.edu.cn (X.D.); 2 State Key Laboratory of Chemical Resource Engineering, Beijing Lab of Biomedical Materials, Beijing University of Chemical Technology, Beijing 100029, China; 2020400119@buct.edu.cn (M.P.); yinmz@mail.buct.edu.cn (M.Y.); 3 Research Institute of Grain Crops, Xinjiang Academy of Agricultural Sciences, Urumqi 830091, China; liuenliang@cau.edu.cn; 4 Adsen Biotechnology Co., Ltd., Urumqi 830022, China; adslizq@126.com

**Keywords:** nano-delivery system, nano-pesticide, predator, selective toxicity, star polymer

## Abstract

Various nano-delivery systems have been designed to deliver synthetic/botanical pesticides for improved bioactivity. However, the enhanced toxicity of nanocarrier-loaded pesticides may injure the natural enemies, and their selective toxicity should be evaluated before the large-scale application. In this context, a star polymer (SPc)-based cyantraniliprole (CNAP) nano-delivery system was constructed, and its selective toxicity was evaluated using pest *Frankliniella occidentalis* (WFT) and predator *Orius sauteri*. The amide NH of CNAP could assemble with carbonyl groups or tertiary amines of SPc through hydrogen bonds to form CNAP/SPc complex spontaneously. The above self-assembly decreased the particle size of CNAP from 808 to 299 nm. With the help of SPc, the lethal concentration 50 (LC_50_) values of CNAP decreased from 99 to 54 mg/L and 230 to 173 mg/L toward WFTs and *O. sauteri* due to the enhancement of broad-spectrum bioactivity. Interestingly, the toxicity selective ratio (TSR) of CNAP increased from 2.33 to 3.23 with the help of SPc, revealing the higher selectivity of SPc-loaded CNAP. To our knowledge, it was the first successful exploration of the selective toxicity of nanocarrier-loaded pesticides, and the higher selective toxicity of SPc-loaded CNAP was beneficial for alleviating the negative impacts on predators.

## 1. Introduction

Nano-delivery systems have been widely studied as transport vehicles for various drugs due to their ability to increase the local accessibility to the target side and enhance bioactivity [1,2,3,4,5,6]. Nanocarriers are materials with at least one dimension in the nanoscale range, which give them unusual physical and chemical features such as quantum effect, high reactivity and high surface area [7]. Compared to the extensive application in medical field, nanocarrier for agrochemical delivery is a recently developed approach [8,9,10,11,12]. Nanocarriers can be successfully applied to deliver double-stranded RNA (dsRNA), Bt toxin and synthetic/botanical pesticides for combating various plant diseases and pests [13,14,15,16,17,18]. The majority of synthetic pesticides are consisted of hydrophobic active ingredients (AIs), thus various nano-delivery systems have been designed to deliver hydrophobic AIs for improved bioactivity, targeted-delivery, controlled-release and enhanced stability characteristics [19,20,21,22].

In recent years, a star polymer (SPc) has been designed and constructed as a gene/pesticide nanocarrier for efficient delivery [23]. The SPc-loaded cargo can be efficiently delivered across the plant/insect cell membrane by activating the clathrin-mediated endocytosis [24,25,26,27,28]. Based on the current publications, the SPc can assemble with several pesticides, such as matrine, osthole and thiamethoxam to form nano-sized pesticides for improved bioactivity, enhanced plant uptake and reduced pesticide residue [29,30,31,32,33]. Thus, the SPc-loaded pesticides have the potential to overcome agricultural, forestry and environmental challenges. Although the working concentration of SPc is relatively safe to predatory ladybirds, the safety and selective toxicity of SPc-loaded pesticides or other nano-pesticides toward predators remain unclear [34]. There are two important issues related to the selective toxicity of SPc-loaded pesticides: (1) whether the SPc increases the toxicity of pesticides against both target pests and non-target predators with the enhancement of broad-spectrum bioactivity, and (2) whether the application of SPc changes the selective toxicity of pesticides.

Western flower thrip (WFTs, *Frankliniella occidentalis*) is a serious insect pest that can cause huge problems in agriculture, horticulture and forestry through feeding, oviposition activity or transmission of plant viruses [35]. Chemical pesticide cyantraniliprole (CNAP) and predator *Orius sauteri* are usually employed to control thrips in actual production [36,37,38,39]. Cyantraniliprole is a second-generation anthranilic diamide insecticide with a broad spectrum registered for suppressing various sucking and chewing insect pests [40,41,42,43,44]. Cyantraniliprole acting on the ryanodine receptor, homotetrameric calcium channels located in the sarco-/endoplasmic reticulum of nerves, can cause the excess release of Ca^2+^ to result in muscle paralysis of insect pests [45,46,47,48]. For CNAP nanometerization, there is only one publication that reports a nanocarrier-loaded CNAP for strong adhesive property on rice leaves and long-term control efficacies against *Cnaphalocrocis medinalis* and *Chilo suppressalis* [49]. However, the selective toxicity of nanoscale CNAP or other nano-pesticides is still not clear.

Previous studies have focused on the enhanced bioactivity of SPc-loaded pesticides. However, the selective toxicity of SPc-loaded pesticides is totally unclear. To this context, a SPc-based CNAP nano-delivery system was constructed and taken as an example to evaluate the selective toxicity of nanopesticide. The characteristic and self-assembly mechanism of CNAP/SPc complex was investigated by determining the pesticide loading content of SPc, testing the interaction between SPc and CNAP and analyzing the particle size and morphology of CNAP/SPc complex. Then, the lethal concentration 50 (LC_50_) values of CNAP/SPc complex were determined toward WFTs and *O. sauteri* through insecticide-impregnated filter method. Finally, the selective toxicity of CNAP/SPc complex and CNAP alone was analyzed and compared by calculating the selective toxicity ratio (STR) and safety coefficient (SC). To our knowledge, it is the first attempt to analyze the selective toxicity of nanocarrier-loaded pesticides, which is beneficial for not only understanding the enhancement of broad-spectrum bioactivity of nano-pesticides, but also providing a theoretical basis for safe application of nano-pesticides.

## 2. Materials and Methods

### 2.1. Chemical Reagents

Pure CNAP (≥94%) was bought from FMC Corporation (Shanghai, China). For SPc synthesis, the N,N,N’,N’,N”-Pentamethyl diethylenetriamine (PMDETA, 98%) and CuBr (≥99%) were bought from Sigma-Aldrich (Saint Louis, MO, USA), the 2-bromo-2-methylpropionyl bromide and triethylamine were bought from Heowns BioChem Technologies (Tianjin, China), and the 2-(Dimethyl amino) ethyl methacrylate (DMAEMA, 99%) was bought from Energy Chemical (Shanghai, China). Other chemical agents were bought from Beijing Chemical Works (Beijing, China).

### 2.2. SPc Synthesis and Preparation of CNAP/SPc Complex

The SPc was synthesized through two steps according to the method described by Li et al. [23]. The CNAP and SPc were dissolved in methanol and ddH_2_O, respectively. The CNAP was mixed with SPc, and the mixture was incubated for 15 min at room temperature. The CNAP could spontaneously assemble with SPc to form CNAP/SPc complex.

### 2.3. Loading Capacity Measurement

The loading capacity of SPc toward CNAP was measured using the freeze-drying method according to the method described by Wang et al. [33]. The 100 mg of CNAP and SPc were dissolved in 25 mL of methanol and 75 mL of double-distilled water (ddH_2_O), respectively. Two solutions were mixed, and the mixture was dialyzed using the regenerated cellulose with a molecular weight cut off of 2000 Da (Shanghai Yuanye Bio-Technology Co., Shanghai, China) for 12 h to exclude the excess CNAP. The CNAP/SPc complex was freeze-dried using a lyophilizer (Beijing Songyuanhuaxing Technology Development Co., Beijing, China) and weighed. The pesticide loading content (PLC) was calculated as PLC (%) = weight of CNAP loaded in complex/weight of CNAP loaded complex × 100%. Each treatment was done in triplicate.

### 2.4. Isothermal Titration Calorimetry (ITC) Assay

The 1 mL of CNAP (0.0138 × 10^−3^ mol/L) was titrated with 250 µL SPc water solution (1 × 10^−3^ mol/L) in Nano ITC (TA Instruments Waters, Newcastle, DE, USA). The heats of interaction during each injection were calculated by integrating each titration peak using the Origin software v. 7.0 (OriginLab Co., Northampton, MA, USA). The test temperature was 25 °C, and ΔG was calculated using the formula of ΔG = ΔH − TΔS.

### 2.5. Particle Size Measurement and Complex Morphology Characterization

The CNAP was dissolved in ethanol, mixed with SPc at the mass ratio of 1:1 and 1:3.03 and diluted with ddH_2_O to prepare the CNAP/SPc complex (0.5 mg/mL). The particle sizes of CNAP and CNAP/SPc complex were measured using a Particle Sizer and Zeta Potential Analyzer (Brookhaven NanoBrook Omni, New York City, NY, USA) at 25 °C. Each treatment included 3 independent samples. The morphological characteristics of CNAP and CNAP/SPc complex at the mass ratio of 1:1 were further examined using a scanning electron microscope (SEM) (JSM-7500F, Tokyo, Japan). A few microliters of above samples were dropped on the surface of silica, dried naturally and coated with a thin layer of platinum for 30 s with ETD-800 sputter coater (Beijing Elaborate Technology Development, Ltd., Beijing, China) before observation.

### 2.6. Bioassay of SPc-Loaded CNAP toward WFTs

The western flower thrips (*F.* *occidentalis*) were collected from organic cucumbers in Beicaiyuan Agricultural Science and Technology Development Co. (Beijing, China), and fed on organic long beans in Organic Agricultural Technology Research Center (China Agricultural University) for nine years. The thrips were pesticide-sensitive strains and maintained at 25 ± 1 °C, 80 ± 10% relative humidity and a 14 L: 10 D photoperiod.

The 10 mg of CNAP was dissolved in detergent TritonX-100 (0.1%), and then mixed with SPc at the mass ratio of 1:1 to prepare the CNAP/SPc complex at the concentrations of 0.2, 0.5 and 2 mg/mL. The solution state of CNAP/SPc complex was compared with CNAP alone to illustrate whether the SPc could improve the solubility of CNAP. Meanwhile, the bioassay of SPc-loaded CNAP and CNAP alone was performed according to the national standard (Guideline for laboratory bioassay of pesticides part 8: insecticide-impregnated filter method). The 1 mL of CNAP/SPc complex (12.44, 24.88, 49.75, 99.5 and 199 mg/L) and CNAP (12.5, 25, 50, 100 and 200 mg/L) was dripped on the qualitative filter paper (9 cm diameter), and the filter paper was air-dried. SPc at a concentration of 199 mg/L and TritonX-100 were applied as controls. The 2nd instar nymphs of WFTs were released on the filter paper and removed to a clean dish at 1 h after the treatment. Thrips that did not move when pushed gently with a brush were scored as dead at 24 and 48 h after the treatment. Each treatment contained 10 thrips and was repeated 10 times.

### 2.7. Bioassy of SPc-Loaded CNAP toward Orius sauteri

The adults of *O. sauteri* were bought from Kuoye Biology Co. (Beijing, China) and fed on WFTs during the experiment. The bioassay of SPc-loaded CNAP and CNAP alone was performed toward *O. sauteri* adults according to insecticide-impregnated filter method similarly as above. The applied concentrations of CNAP/SPc complex and CNAP were same with above experiment. The SPc at the concentration of 199 mg/L and TritonX-100 was applied as controls. The mortality was recorded at 24 and 48 h after the treatment. Each treatment contained 10 *O. sauteri* adults, which was repeated 10 times.

### 2.8. Data Analysis

The statistical analysis was performed using the SPSS 19.0 software (SPSS Inc., New York, NY, USA). The descriptive statistics were shown as the mean value and standard errors of the mean. The Tukey HSD test was used to analyze the particle size at the *p* = 0.05 level of significance.

Concentration-mortality data were analyzed to obtain the lethal concentration 50 (LC_50_) using POLOPlus version 2.0 (LeOra Software, CA, USA, 2002) [50]. Efficiency ratio was given as the ratio of the CNAP LC_50_ to complex LC_50_.

The safety of CNAP/SPc complex was analyzed by calculating the selective toxicity ratio (STR) and safety coefficient (SC) [51,52,53]. STR was calculated as STR = predator’s LC_50_ ÷ pest’s LC_50_. The pesticide can be classified as selective pesticide when STR > 1. SC was calculated as SC = predator’s LC_50_ ÷ recommended concentration of pesticide for field application. The pesticide can be classified as medium risk when 0.5 < SC ≤ 5. The recommended concentration of CNAP is 111.33–133.33 mg/L for controlling thrips in actual production.

## 3. Results

### 3.1. SPc Synthesis and Its Loading Capacity

**Figure 1:**
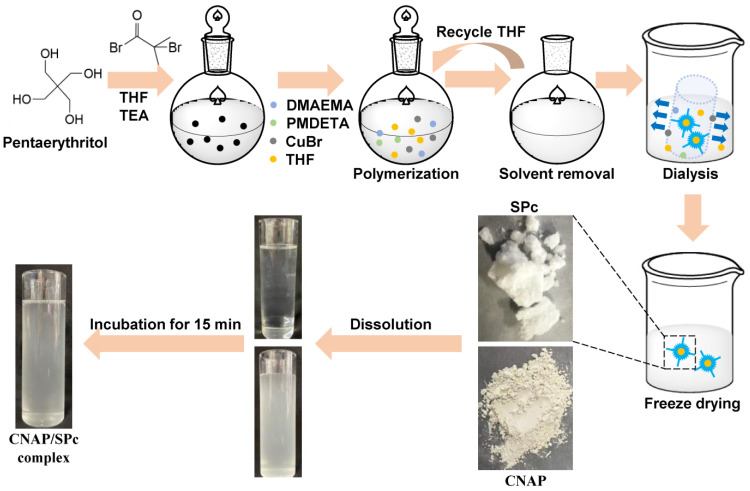
Synthesis route of SPc and preparation of CNAP/SPc complex.

The synthesis route of SPc includes two reaction steps. As shown in Figure 1, the 2-bromo-2-methylpropionyl bromide was added into the pentaerythritol solution in dry tetrahydrofuran (THF) and triethylamine (TEA) to obtain the star initiator. The star initiator, DMAEMA and dry THF were added into a flask and degassed by nitrogen, and the CuBr and PMDETA were then added for polymerization. The crude polymer was purified by dialysis, and the white powder of SPc was finally obtained. Furthermore, the THF could be recycled to decrease the production cost of SPc. The 100 mg of CNAP and SPc were dissolved in 25 mL of methanol and 75 mL of double-distilled water (ddH_2_O), respectively. Two solutions were mixed, and the CNAP could spontaneously assemble with SPc to form the CNAP/SPc complex. The mixture was dialyzed to exclude the excess CNAP, and the obtained CNAP/SPc complex was then freeze-dried and weighed. As shown in Table 1, the pesticide loading content was calculated to be 24.79%.

### 3.2. Interaction of CNAP with SPc

**Figure 2:**
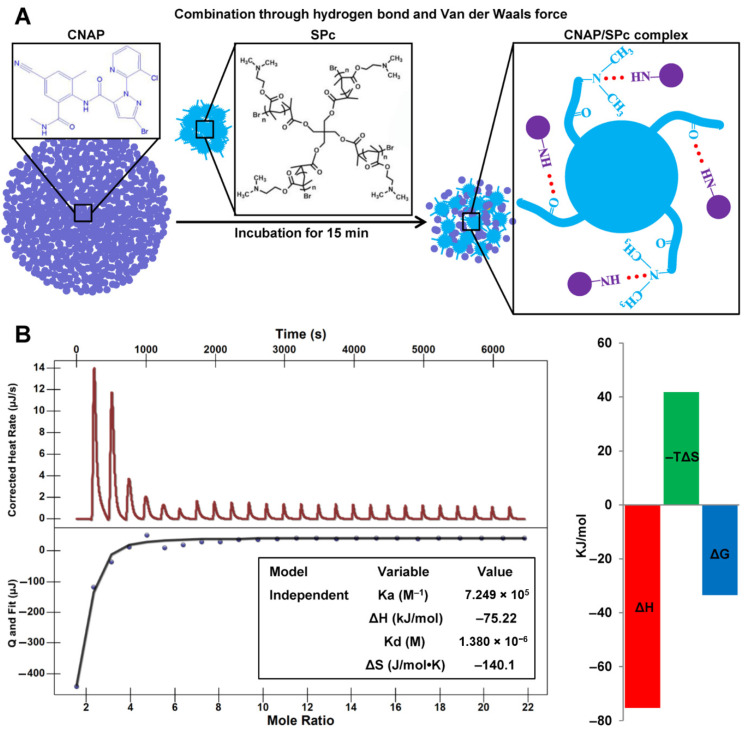
Schematic illustration of CNAP/SPc complex (**A**) and ITC titration of SPc (1 mM) into CNAP solution (0.0138 mM) (**B**).

The CNAP solution was titrated with SPc solution to detect the interaction force between CNAP and SPc. The high affinity constant Ka of 7.249 × 10^5^ M^−1^ and low dissociation constant Kd of 1.380 × 10^−6^ M indicated an effective and strong interaction between CNAP and SPc, and this interaction was automatic due to the negative ΔG value (−33.449 kJ/mol) (Figure 2). The negative values of ΔH (−75.22 kJ/mol) and ΔS (−140.1 J/mol•K) suggested that the self-assembly of CNAP with SPc was through hydrogen bonding and Van der Waals forces. Based on the chemical structures of CNAP and SPc, the putative sites for hydrogen bonding might be the amide NH of CNAP with carbonyl groups or tertiary amines of SPc.

### 3.3. Characterization of CNAP/SPc Complex

**Figure 3:**
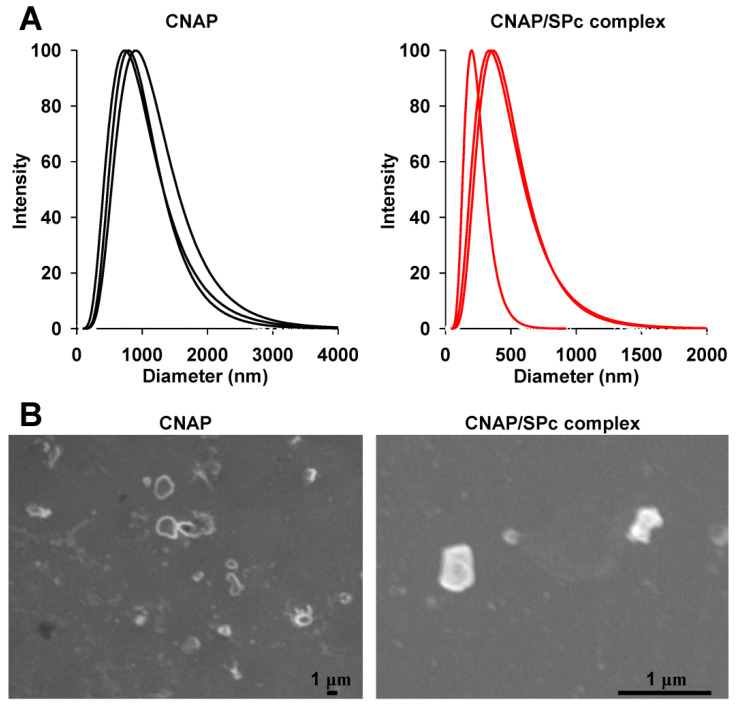
Particle size distributions (**A**) and SEM images (**B**) of CNAP and CNAP/SPc complex at the mass ratio of 1:1.

The dynamic light scattering (DLS) was used to measure the particle size of CNAP/SPc complex (Table 2 and Figure 3A). The CNAP could aggregate into large particles with mean diameter of 808 nm, whereas the assembly of CNAP/SPc complex at the mass ratio of 1:1 disturbed the self-aggregated structure of CNAP, forming smaller particles (299 nm). Furthermore, the particle size of CNAP/SPc complex was not significantly changed at various mass ratios, revealing that only a small amount of SPc could reduce the particle size of CNAP to nanoscale. This conclusion was also supported by the results of scanning electron microscope (SEM) (Figure 3B). The particle sizes of both CNAP and CNAP/SPc complex varied greatly among different particles, and the CNAP/SPc complex self-aggregated into smaller particles with irregular shape compared to CNAP alone. The CNAP/SPc complex at the mass ratio of 1:1 was employed for the following experiments.

### 3.4. Toxicity of CNAP/SPc Complex against WFTs

**Figure 4:**
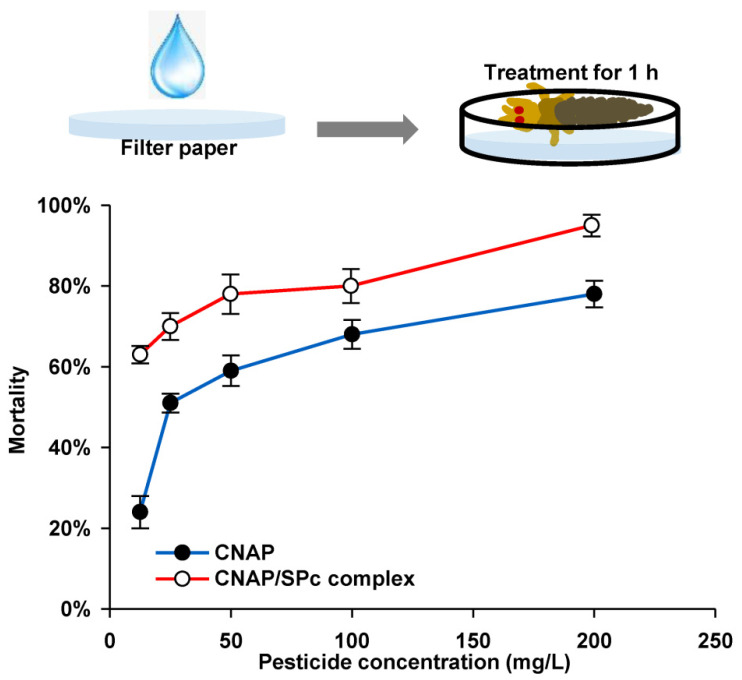
Toxicity of CNAP/SPc complex against 2nd instar nymphs of WFT at 48 h after the treatment. *n* = 10.

The LogP of CNAP is 4.43, and its water solubility is less than 0.1 mg/mL, revealing the hydrophobic character of CNAP (Provided by ChemSrc). Thus, the CNAP was dissolved in detergent TritonX-100 (0.1%) to prepare the CNAP/SPc complex, and the solution state of CNAP/SPc complex was also observed and compared with CNAP alone. Our results revealed that the SPc could not improve the solubility of CNAP (Figure S1 from Supplementary Materials). Meanwhile, the LC_50_ values of CNAP/SPc complex and CNAP alone were determined toward the 2nd instar nymphs of WFTs through the insecticide-impregnated filter method. As expected, the SPc at the highest concentration showed no toxicity toward WFTs. With the help of SPc, the LC_50_ values of CNAP decreased from 98.695 to 53.714 mg/L at 24 h after the treatment (Table 3), and the mortality of thrips was increased by approximately 20% at 48 h after the treatment (Figure 4).

### 3.5. Selective Toxicity of CNAP/SPc Complex against O. sauteri

**Figure 5:**
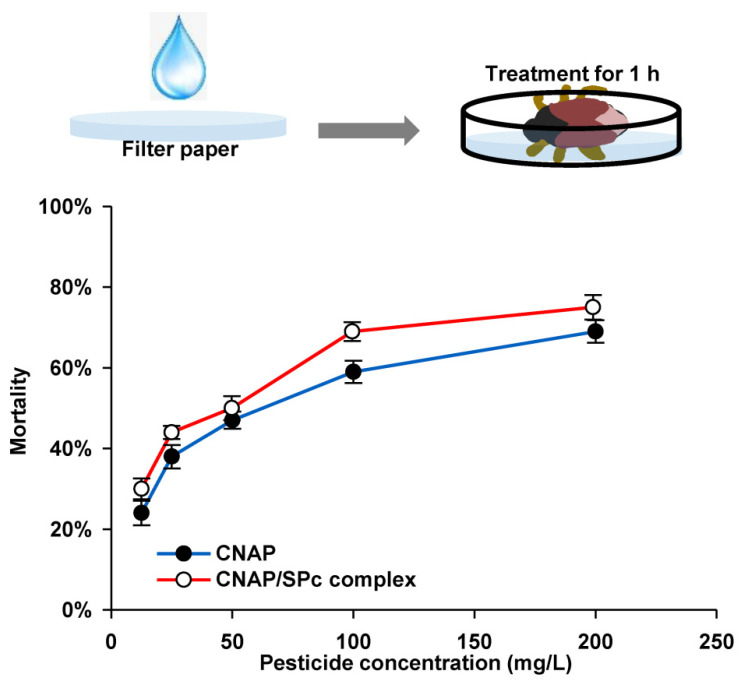
Toxicity of CNAP/SPc complex against the adults of *Orius sauteri* at 48 h after the treatment. *n* = 10.

The potential negative effects of CNAP/SPc complex should be evaluated toward predators before the large scale field application. Thus, the LC_50_ values of CNAP and CNAP/SPc complex were determined toward *O. sauteri* using the similar bioassay method. The SPc also exhibited negligible toxicity toward *O. sauteri*. After the complexation with SPc, the LC_50_ values of CNAP decreased from 229.662 to 173.437 mg/L at 24 h after the treatment (Table 4), and the toxicity of SPc-loaded CNAP was slightly improved due to the enhancement of broad-spectrum bioactivity (Figure 5). The toxicity of SPc-loaded CNAP was improved against both target pests and non-target predators, but whether the selective toxicity of CNAP changed after the complexation with SPc was crucial. The toxicity-selective ratio (TSR) was firstly calculated to analyze the selective toxicity of SPc-loaded CNAP. The TSR of CNAP increased from 2.33 to 3.23 with the help of SPc at 24 h after the treatment, indicating the higher selectivity of SPc-loaded CNAP compared to CNAP alone. Furthermore, the recommended concentration of CNAP is 111.33–133.33 mg/L for field application, and the safety coefficient of SPc-loaded CNAP was 1.56–1.30 that was between 0.5 and 5. The CNAP/SPc complex can be classified as medium risk toward *O. sauteri*.

## 4. Discussion

As a gene/pesticide nanocarrier, the SPc can be complexed with various exogenous substances for enhanced delivery and bioactivity [24,25,29,30]. The current study illustrated that the pesticide loading content of SPc toward CNAP was 24.79%, which was higher than those of osthole (17.09%), thiamethoxam (20.63%), monosultap (19.3%), and dinotefuran (17.41%) [30,31,32,33]. The binding affinity of CNAP with SPc was then analyzed using a high-accuracy method ITC [54,55]. The interaction force was analyzed according to the previous interpretation of ITC data [56]. Our results revealed that there was a strong hydrogen bonding and Van der Waals forces between CNAP and SPc, and we deduced the amide NH of CNAP and carbonyl groups or tertiary amines of SPc played an important role in the self-assembly of CNAP/SPc complex. Consistent with previous studies, the SPc can assemble with dinotefuran, monosultap, avermectin and chitosan through hydrogen bond and Van der Waals forces [25,32,33,57]. Meanwhile, the SPc can also combine with thiocyclam, eugenol, thiamethoxam, matrine and osthole through different interaction forces, such as electrostatic interaction and hydrophobic association [25,29,30,31,33]. Different self-assembly mechanisms of pesticide/SPc complexes are beneficial for expanding the application area of SPc, indicating that the SPc may be a universal adjuvant for pesticide delivery.

Compared to CNAP alone, the complexation of CNAP with SPc formed smaller particles with irregular shape, and the morphology and particle size of SPc-loaded pesticides are not only related to the chemical structures of pesticide and SPc, but also the interaction between pesticide and SPc. Meanwhile, the nanometerization of SPc-loaded CNAP in aqueous solution was similar to our previous studies that the complexation with SPc could decrease the particle sizes of insecticides, such as avermectin, dinotefuran and matrine down to nanoscale [29,32,57]. For instance, the particle size of matrine can be reduced from 858 to 9 nm with the help of SPc, and self-assembly of thiamethoxam/SPc complex can decrease the particle size of thiamethoxam from 576 to 116 nm [29,31]. The nanometeriztion of SPc-loaded pesticide can not only increase the contact area of pesticides to target pests for enhanced contact toxicity, but also improve the plant uptake and systemic transmission in plants for enhanced stomach toxicity. Furthermore, the current study demonstrated that only a small amount of SPc could reduce the particle size of CNAP to nanoscale, and this property was beneficial for reducing the application amount of SPc, which revealed that the SPc was fit for the large scale application in field.

The toxicity of CNAP has been analyzed against the nymphs and adults of WFTs using the oral feeding method [38]. For nymphs, the LC_50_ values of field strains range from 33.4 to 109.2 mg/L with a low natural variability of 3.3 folds. For adults, the LC_50_ values of CNAP range from 536 to 2415 mg/L with a slight natural variability of 4.5 folds. The sublethal effects of CNAP have been observed, such as reduced fecundity, fertility, feeding, oviposition and mating. Furthermore, some antifeedant responses have also been also observed in electrical penetration graphing studies, and CNAP can reduce the probability of tomato spotted wilt virus infection in field-grown peppers [37]. In the current study, the toxicity of CNAP was significantly improved against WFTs with the help of SPc. Similar to previous studies, the LC_50_ values of osthole decrease from 49 to 34 mg/L toward green peach aphids and from 332 to 270 mg/L toward two-spotted spider mites with the help of SPc [30]. Furthermore, with the help of SPc, the LC_50_ value of pure thiocyclam decreases from 532 to 221 mg/L toward green peach aphids [33]. The potential mechanism of enhanced contact toxicity may be the CNAP nanometerization by the nano-delivery system, which leads to enlarged contact area to target pests and improved penetration across the insect cuticle. Previous studies have confirmed that the SPc-loaded dsRNA can penetrate the insect cuticle for efficient RNA interference [24,26,27].

The potential negative effects of pesticides should be evaluated toward predators, pollinators and environmental microbiota to guarantee their safety use. Xiao et al. [58] has tested the toxicity of three common insecticides against predatory enemies and parasitoids, and the predatory *O. sauteri* exhibits stronger tolerance than most tested insects with higher LC_50_ values. In addition, a highly virulent entomopathogenic fungus (*Beauveria bassiana*) toward WFTs is not insecticidal against *O. sauteri* [59]. In the current study, the bioactivity of SPc-loaded CNAP was also slightly enhanced toward *O. sauteri*, which is consistent with the previous study that the immersion of SPc-loaded dinotefuran leads to higher mortalities of both green peach aphids and predatory lady beetles [32]. Interestingly, the TSR was increased with the help of SPc, revealing the higher selectivity of CNAP/SPc complex. Furthermore, the CNAP/SPc complex can be classified as medium risk toward *O. sauteri* according to its safety coefficient. A recent publication has determined the selective toxicity of eight neonicotinoid insecticides toward WFTs and *O. sauteri*, and the LC_50_ values of most tested insecticides are higher toward WFTs than *O. sauteri*, suggesting the higher toxicity toward *O. sauteri* [39]. Thus, the CNAP/SPc complex or CNAP alone was relatively safer toward *O. sauteri* compared to neonicotinoid insecticides. The SPc-loaded matrine and predatory ladybirds have been co-applied to suppress the green peach aphids, which can overcome the slow-acting property of ladybirds [34]. The co-application of CNAP/SPc complex and *O. sauteri* may be suitable for efficient control of WFTs, which will be further tested for reducing the pesticide application.

## 5. Conclusions

In this work, *F. occidentalis* -*O. sauteri* was taken as an example to evaluate the selective toxicity of CNAP nano-delivery system. The amide NH of CNAP could interact with carbonyl groups or tertiary amines of SPc through hydrogen bond to form CNAP/SPc complex spontaneously. Above self-assembly could reduce the particle size of CNAP from 808 down to 299 nm. Compared to CNAP alone, the SPc-loaded CNAP exhibited stronger toxicity against both WFTs and *O. sauteri* due to the enhancement of broad-spectrum bioactivity, and the LC_50_ values of CNAP decreased from 99 to 54 mg/L and 230 to 173 mg/L toward WFTs and *O. sauteri* with the help of SPc at 24 h after the treatment, respectively. For selective toxicity analysis, the TSR of CNAP increased from 2.33 to 3.23 with the help of SPc at 24 h after the treatment, indicating the higher selectivity of SPc-loaded CNAP. Furthermore, the safety coefficient of SPc-loaded CNAP was 1.56–1.30, suggesting the medium risk toward *O. sauteri*. To our knowledge, it is the first case to analyze the selective toxicity of nanocarrier-loaded pesticides, and the higher selective toxicity of SPc-loaded CNAP was beneficial for alleviating the negative impacts on predators.

## Figures and Tables

**Table 1:** Loading capacity of SPc toward CNAP using freeze-drying method.

Sample Number	Weight of Applied CNAP (mg)	Weight of Applied SPc (mg)	Weight of CNAP-Loaded Complex (mg)	Weight of CNAP Loaded in Complex (mg)	Pesticide Loading Content (%)	Average Pesticide Loading Content (%)
1	10.0	10.0	13.5	3.5	25.93	24.79 ± 0.87
2	10.0	10.0	13.0	3.0	23.08	
3	10.0	10.0	13.4	3.4	25.37	

Mean ± SE.

**Table 2:** Reduced particle size of SPc-loaded CNAP.

Formulation	Sample Number	Mass Ratio	Polydispersity	Size (nm)	Average Size (nm)
CNAP	1	-	0.211	789.71	807.86 ± 49.34
	2		0.230	900.94	
	3		0.286	732.94	
CNAP/SPc complex	1	1:1	0.290	335.94	298.92 ± 50.79
	2		0.153	198.49	
	3		0.238	362.34	
	1	1:3.03	0.251	251.45	289.01 ± 24.40
	2		0.242	280.83	
	3		0.235	334.76	
*F_2,6_* = 47,094, *p* < 0.001					

The mean ± SE was analyzed by Tukey HSD test (*p* < 0.05).

**Table 3:** Toxicity of CNAP and CNAP/SPc complex against 2nd instar nymphs of WFT at 24 h after the treatment.

Formulation	LC_50_ (mg/L) (95% Confidence Limits)	Slope ± SE	χ^2^(df) ^a^	Efficiency Ratio
CNAP	98.695 (74.040–146.350)	0.968 ± 0.141	15.480 (48)	1.837
CNAP/SPc complex	53.714 (42.517–68.443)	1.146 ± 0.142	19.357 (48)	

^a^ Chi-square value and degrees of freedom (df) were calculated by PoloPlus.

**Table 4:** Toxicity of CNAP and CNAP/SPc complex against adults of *Orius sauteri* at 24 h after the treatment.

Formulation	LC_50_ (mg/L) (95% Confidence Limits)	Slope ± SE	χ^2^(df) ^a^	Efficiency Ratio
CNAP	229.662 (155.278–437.831)	1.012 ± 0.153	13.736 (48)	1.324
CNAP/SPc complex	173.437 (119.378–319.255)	0.918 ± 0.145	15.978 (48)	

^a^ Chi-squared value and degrees of freedom (df) were calculated by PoloPlus.

## Data Availability

All data in this study will be available from the corresponding author upon reasonable request.

## Supplementary Materials

The following are available online at https://www.mdpi.com/article/10.3390/nano12142419/s1, Figure S1: Solubility test of CNAP/SPc complex.
