# Supplementary material for: A Preparation Method of Nano-Pesticide Improves the Selective Toxicity toward Natural Enemies
Source: Nanomaterials (Basel). 2022 Jul 14;12(14):2419. doi: 10.3390/nano12142419 (PMC9323491; doi:10.3390/nano12142419)
Supplement: Supplementary file 1 [file nanomaterials-12-02419-s001.zip › nanomaterials-1793442-supplementary.pdf]

nanomaterials

Supplementary Materials

A Preparation Method of Nano-Pesticide Improves the Selective Toxicity toward Natural Enemies

Shuo Yan [1], Na Gu [1], Min Peng [2], Qinhong Jiang [1], Enliang Liu [3], Zhiqiang Li [4], Meizhen Yin [2], Jie Shen [1], Xiangge Du [1] and Min Dong [1,*]

[1] Department of Plant Biosecurity and MARA Key Laboratory of Surveillance and Management for Plant Quarantine Pests, College of Plant Protection, China Agricultural University, Beijing 100193, China; yanshuo2011@foxmail.com (S.Y.); s20193192665@cau.edu.cn (N.G.); j.yucheng@outlook.com (Q.J.); shenjie@cau.edu.cn (J.S.); duxge@cau.edu.cn (X.D.)
[2] State Key Laboratory of Chemical Resource Engineering, Beijing Lab of Biomedical Materials, Beijing University of Chemical Technology, Beijing 100029, China; 2020400119@buct.edu.cn (M.P.); yinmz@mail.buct.edu.cn (M.Y.)
[3] Research Institute of Grain Crops, Xinjiang Academy of Agricultural Sciences, Urumqi 830091, China; liuenliang@cau.edu.cn
[4] Adsen Biotechnology Co., Ltd., Urumchi 830022, China; adslizq@126.com
* Correspondence: dongmin@cau.edu.cn

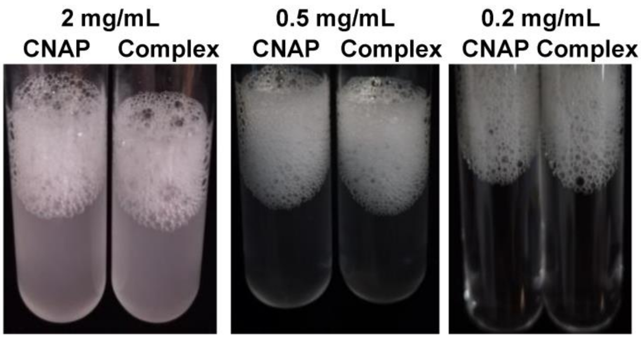

Figure S1. Solubility test of CNAP/SPc complex.
